# Single-cell transcriptome analysis reveals heterogeneity and convergence of the tumor microenvironment in colorectal cancer

**DOI:** 10.3389/fimmu.2022.1003419

**Published:** 2023-01-04

**Authors:** Siyuan Xie, Yangke Cai, Delong Chen, Yu Xiang, Wen Cai, Jianshan Mao, Jun Ye

**Affiliations:** ^1^ Department of Gastroenterology, The Second Affiliated Hospital, Zhejiang University School of Medicine, Hangzhou, Zhejiang, China; ^2^ Department of Cardiology, The Second Affiliated Hospital, Zhejiang University School of Medicine, Hangzhou, Zhejiang, China; ^3^ Department of Gastroenterology, Huzhou Central Hospital, Huzhou, Zhejiang, China

**Keywords:** ScRNA-seq, colorectal cancer, tumor microenvironment, CIBERSORTx, prognosis

## Abstract

**Introduction:**

Colorectal cancer (CRC) ranks second for mortality and third for morbidity among the most commonly diagnosed cancers worldwide. We aimed to investigate the heterogeneity and convergence of tumor microenvironment (TME) in CRC.

**Methods:**

We analyzed the single-cell RNA sequencing data obtained from the Gene Expression Omnibus (GEO) database and identified 8 major cell types and 25 subgroups derived from tumor, para-tumor and peripheral blood.

**Results:**

In this study, we found that there were significant differences in metabolic patterns, immunophenotypes and transcription factor (TF) regulatory patterns among different subgroups of each major cell type. However, subgroups manifested similar lipid metabolic patterns, immunosuppressive functions and TFs module at the end of the differentiation trajectory in CD8+ T cells, myeloid cells and Fibroblasts. Meanwhile, TFs regulated lipid metabolism and immunosuppressive ligand-receptor pairs were detected by tracing the differentiation trajectory. Based on the cell subgroup fractions calculated by CIBERSORTx and bulk RNA-sequencing data from The Cancer Genome Atlas (TCGA), we constructed an immune risk model and clinical risk model of CRC which presented excellent prognostic value.

**Conclusion:**

This study identified that the differentiation was accompanied by remodeling of lipid metabolism and suppression of immune function, which suggest that lipid remodeling may be an important trigger of immunosuppression. More importantly, our work provides a new perspective for understanding the heterogeneity and convergence of the TME and will aid the development of prognosis and immunotherapies of CRC patients.

## 1 Introduction

Colorectal cancer (CRC) accounts for about 10% of all malignant neoplasms in humans which is the third most common cancer worldwide and its mortality rate (9.4%) was the second highest among malignancies, only after lung cancer. As of 2020, more than 935,000 people worldwide died from CRC or its complications (1). The traditional mode of surgery combined with chemoradiotherapy has not achieved the ideal curative effect (2). In this context, immunotherapy emerged and quickly became the main treatment mode for a variety of tumors, including CRC, and achieved long-term and sustained remission in a small number of patients, however, the majority of patients did not achieve long-term tumor control after a temporary immune response. This indicates that although immunotherapy has great prospects in tumor treatment, there are still considerable deficiencies at present. We believe that the fundamental solution is to improve the understanding of the tumor microenvironment (TME).

TME plays an important role in the occurrence, development and metastasis of tumors, including not only tumor cells, but also immune cells, stromal cells, cytokines, extracellular matrix and other extracellular components (3). There have been extensive studies on the heterogeneity of TME, most of which focus on the heterogeneity of tumor cells, but the heterogeneity of immune cells and stromal cells is still insufficient. In recent years, more and more studies have confirmed that tumor Infiltrating T lymphocytes (TILs) will gradually differentiate into a dysfunctional state which is known as exhaustion under long-term antigen stimulation, which is one of the main obstacles to anti-tumor immunotherapy in the process of tumor development. The exhausted CD8+ T cells (Tex) were characterized by progressive and hierarchical loss of cytokine production, high co-expression of inhibitory receptors (programmed cell death 1 (PD-1), lymphocyte activation gene 3 protein (LAG3), T cell immunoreceptor with immunoglobulin and ITIM domain (TIGIT), etc.), altered expression of key transcription factors and metabolic derangement (4). Meanwhile, immune checkpoint inhibitor therapy has achieved unprecedented clinical success in a variety of cancers particularly PD-1 antibodies (5). T-cell receptor (TCR) persistent activation, transcription factors (including Signal transducer and activator of transcription 3 (STAT3), STAT4, Nuclear factor of activated T-cells, cytoplasmic 1 (NFATC1) and Blimp-1) and epigenetic components (including DNA methylation) were reported to regulate the expression of immune checkpoints (6–8). However, the metabolic reprogramming was associated with the development and maintenance of Tex while the detailed mechanism remained unclear. In addition, tumor associated macrophages (TAMs) and cancer associated fibroblasts (CAFs) have also been reported as potential targets of tumor immunotherapy. They are heterogeneous cell types which contributed to malignancy through production of angiogenic growth factors, extracellular matrix (ECM) remodeling, and immunosuppression (9, 10). The immunotherapy targeted TAMs has been applied in clinic while the minimal monotherapy efficacy was observed (11). Similarly, altered metabolism in the development of TAMs and CAFs has also been reported while the specific mechanism remains unknown. Notably, investigation of heterogeneity and convergence of above cell types in TME may contribute to clarify the relationship between immunosuppression and metabolic remodeling and find potential therapeutic targets.

Single-cell RNA sequencing (scRNA-seq) is a huge innovation and technological progress in the field of life science. It provides us with gene expression information at the level of individual cells and is an indispensable tool to unravel cellular heterogeneity (12). In this study, we obtained scRNA-seq data from the public database, re-identified and annotated cell populations and constructed cell differentiation tracks, identified multiple cell subpopulations, and found that different types of cells always showed similar phenotypes at the end of their differentiation tracks, which was called convergence. While recent studies have attempted to fully elucidate the TME heterogeneity identified by scRNA-seq in human cancers, there are significant deficiencies in the elucidations of convergence in TME. In this study, we not only focus on the heterogeneity of TME, but also identified the convergence and detected common targets of different cell types which may be potential therapeutic targets and help improve the treatment strategy and clinical prognosis of patients with CRC.

## 2 Materials and methods

### 2.1 Data acquisition

The scRNA-seq profiles included 10,398 cells from 10 human CRC samples (accession number GSE146771) (13), which were obtained from the Gene Expression Omnibus (GEO, http://www.ncbi.nlm.nih.gov/geo/) database. This dataset contains 5169 cells from tumor cores, 2400 cells from paratumor tissues and 2829 cells from peripheral blood, performed using the SMART-seq2 platform. Normalized matrix files for the dataset were downloaded. The bulk RNA-seq data of CRC samples, including 398 tumor samples and 39 normal samples, were obtained from the The Cancer Genome Atlas (TCGA) database (https://portal.gdc.cancer.gov/). We excluded samples with an overall survival (OS) time< 7 days or insufficient clinical information regarding age, gender, or TNM stage.

### 2.2 Processing of the CRC scRNA-seq data

The Seurat package in R 4.0.3 was used for quality control (QC) (14). The quality standards were as follows: 1) genes detected in< 3 cells were excluded; 2) cells with< 50 total detected genes were excluded; 3) cells with ≥ 5% of mitochondria-expressed genes were excluded. For the remaining cells, cell-cycle scores were calculated using Seurat’s CellCycleScoring function since the cell cycle phase effect was observed. Batch effects among the patients had already been eliminated by the data donator. The gene expression matrices were further normalized to RNA counts, mitochondrial percentages, and cell cycle scores using the top 3000 variable genes. PCA was used to calculate the significantly available principal components (PCs). We then applied the t-distributed stochastic neighbor-embedding (tSNE) algorithm for dimensionality reduction with 20 initial PCs to perform cluster classification analyses across all cells (15).

### 2.3 Cell type recognition

We performed differential expression analysis among all genes within cell clusters using Seurat’s FindAllMarkers function to identify the marker genes in each cluster (16). An adjusted P-value< 0.05, expression percentage > 0.25, and | log2 [fold change (FC)] | > 0.25 were considered as cutoff criteria for identifying marker genes (**Table S1**). Subsequently, different cell clusters were determined and annotated by the singleR package according to the composition patterns of the marker genes and were then manually verified and corrected with the CellMarker database. The malignant cells were annotated by correlation with the data donator’s cell annotation.

### 2.4 Pseudotime trajectory analysis

Single-cell pseudotime trajectories were constructed using the Monocle 2 algorithm, an R package designed for single-cell trajectories by Qiu et al (17). This algorithm applies a machine learning technique to reduce the high-dimensional expression profile to a low-dimensional space, visualized as a tSNE plot. Single cells were projected onto this space and ordered into a trajectory with branch points. The dynamic expression heatmap was constructed using the plot_pseudotime_heatmap function. In addition, differential expression analysis between branches was performed using the plot_genes_branched_heatmap function.

### 2.5 Functional enrichment analysis

Differentially expressed genes (DEGs) analysis was performed using Seurat’s FindMarkers function. The following cutoff threshold values were used: adjusted P-value< 0.05 and |log2 [FC]| >1. The DEGs were loaded into Metascape (http://metascape.org), a tool for gene list enrichment analysis (18).

The Gene Set Variation Analysis (GSVA) algorithm was performed to explore the activity variation of biological process and pathways in each cell types. Gene Oncology gene sets “c2.all.v7.4.symbols.gmt” and Kyoto Encyclopedia of Genes and Genomes sets “c5.all.v7.4.symbols.gmt” from Molecular Signatures Database (MSigDB, http://www.gsea-msigdb.org), which were used for functional analyses. The GSVA analysis was performed in R 4.0.3 to calculate the enrichment score of the pathways in each cell and when the P-value was less than 0.05, the enriched gene set was considered to be statistically significant.

### 2.6 Cell-cell communication analysis

CellChat is a novel toolkit used to infer intercellular communication networks from scRNA-seq data quantitatively (19). Based on the ligand-receptor interactions database for human and pattern recognition approaches, CellChat can predict major signaling inputs and outputs for cells and establish how those cells and signals coordinate their functions. Ligand-receptor pairs with a P-value< 0.05 were filtered to evaluate the relationship between different cell types.

### 2.7 Gene regulatory network analysis

We used SCENIC (Aibar et al., 2017) (20), an algorithm that can reconstruct transcriptional states and regulatory networks from scRNA-seq data, to evaluate the gene regulatory networks relating to TFs and regulons in individual cells. The gene expression matrix was input into SCENIC and a co-expression matrix was constructed using GENIE3. Direct binding by DNA-motif analysis was identified based on a motif dataset (hg19-500bp-upstream-7species.mc9nr.feather, hg19-tss-centered-10kb-7species.mc9nr.feather) to construct regulons for each TF. Finally, regulon activity was analyzed using AUCell (Area under the Curve), where a default threshold was applied to binarize the specific regulons. Regulon modules were then identified based on the Connection Specificity Index (CSI) to confirm specific associating partners (21). Hierarchical clustering with Euclidean distance was then performed to identify different regulon modules. We then used 0.65 as a cutoff to construct the regulon association network, to investigate the relationship between different regulons.

### 2.8 Correlation with bulk RNA-seq data

CIBERSORTx is a new machine learning method developed from CIBERSORT for estimating the abundance of cell clusters in bulk RNA-seq data (22). This tool was used to digitally purify the transcriptome of individual cell clusters from the bulk data without isolating single cells. We extracted the transcripts per million (TPM) normalization datasets of selected cell types including CD8+ T cells, myeloid cells, fibroblasts and epithelial cells to create the signature matrix in 1000 permutations and without batch correction. Then we separated the CRC patients from TCGA database into training and testing cohorts according to a 1:1 ratio using a randomization method based on survival status and used CIBERSORTx to estimate the fraction of each cell cluster in training and testing cohorts respectively. Notably, the bulk RNA-seq data from TCGA was first normalized to TPM values. Furthermore, stepwise multivariate Cox regression was applied to select the optimal coefficient for each cell cluster to construct the risk model in training cohort. The riskscore were then divided into “high risktype” and “low risktype” according to the median risk score which equaled 1.263 in the training cohort. The formula for the model is as follows:


$$\text{Riskscore}=\sum_{i=1}^{n}Coef_{i}*Fraction_{i}$$

Finally, we incorporated the riskscore, TNM stage, gender, and age to construct a clinical risk model using stepwise multivariate Cox regression to construct clinical risk model in the training cohort. The clinical riskscores was then divided into “high clinical risktype” and “low clinical risktype” according to the median risk score which equaled 0.900 in the training cohort. The formula for the model is as follows:


$$\text{Clinical Riskscore}=\sum_{i=1}^{n}Coef_{i}*Factor_{i}$$

The associations of immune risktype and clinical risk type with OS were analyzed using Kaplan-Meier (KM) survival analysis, with receiver operating characteristic (ROC) curve analysis used to verify the sensitivity and specificity of the model for the training cohort. The immune risk model and clinical risk model was then applied to the testing cohort, and the reliability of the model was verified by KM curve and ROC curve analyses.

### 2.9 Statistical analyses

Statistical analyses were conducted using R software (version 4.0.3; R Foundation for Statistical Computing, Vienna, Austria). All statistical tests were two-sided, with P-values< 0.05 considered statistically significant.

## 3 Results

**Figure 1:**
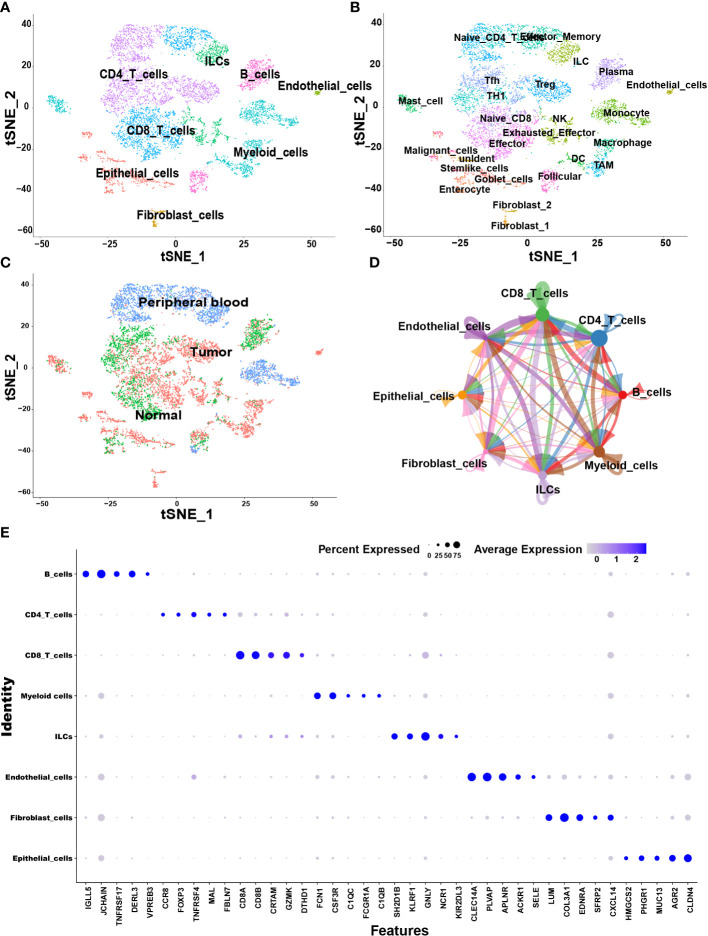
Overview of single cells derived from tumors, adjacent tumor tissues, and peripheral blood of CRC patients. **(A–C)** tSNE plots of all the single cells color-coded for **(A)** eight major cell types, **(B)** 25 sub-cell types, **(C)** tissue origins (tumor, adjacent to tumor or blood). **(D)** Interaction network among major cell types constructed by CellChat; circle sizes represent interaction weights; the thicker line indicates more weight and strength of the interactions between variable major cell types. **(E)** Bubble heatmap showing top five marker genes of eight major cell types. Dot size indicates fraction of expressing cells, colored according to expression levels.

The samples, including tumor, Para-tumor and blood from 10 treatment-naive CRC patients were involved in this study. According to the annotation of SingleR package and CellMarker database, we finally identified 8 major cell types including CD4+ T cells, CD8+ T cells, B cells, myeloid cells, innate lymphoid cells (ILCs), fibroblast cells, endothelial cells, and epithelial cells (**Figure 1A**). Each cell type was extracted and further grouped for annotation, and finally 25 cell subtypes were identified (**Figure 1B**). The top five markers identified by the differences in the main cell types were visualized as a bubble plot (**Figure 1E**). Interestingly, when we traced the tissue origins, it was noted that immune cells, especially Tex, TAMs, dendritic cells (DCs) and fibroblast cells were highly enriched in tumor tissues (**Figures 1C**). To investigate the network of interactions in the TME, we used CellChat to calculate potential ligand-receptor pairs. Network visualization was performed to visualize the interactions (**Figure 1D**). Notably, Tex, macrophages, TAMs, and DCs possessed the most interaction pairs with cells from other lineages, revealing the dominant roles in the TME.

### 3.1 CD8+ T cells

**Figure 2:**
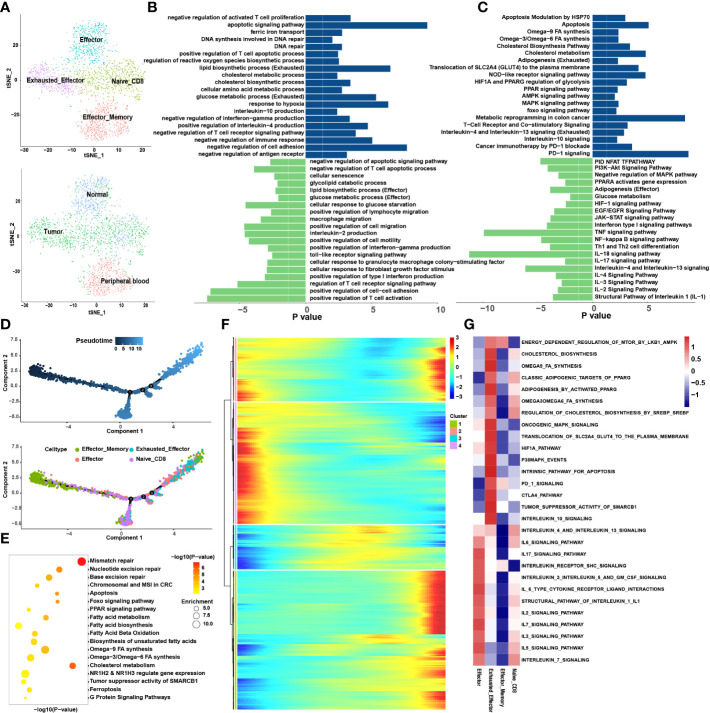
CD8+ T cells tend to exhaust in the tumor microenvironment. **(A)** tSNE plots showing 4 sub-cell types of CD8+ T cells (upper) and their tissue origins (lower). **(B)** GO and **(C)** KEGG pathway enrichment analyses of marker genes of Tex (blue color) and Teff (green color). The height of each barplot shows the log10 of P-value calculated using the Metascape database. **(D)** Differentiation trajectory of CD8+ T cells in CRC, color-coded for pseudotime (upper) and sub-cell types (lower). **(E)** Pseudo-heatmap of genes altered in the differentiation process of CD8+ T cells in CRC, divided into four clusters. **(F)** The bubble plot shows the GO and KEGG pathway enrichment analysis of genes in cluster 1 identified in Pseudo-heatmap using the Metascape database. **(G)** The heatmap illustrates the activity of biological process and signaling pathway in each cell type by GSVA.

**Figure 3:**
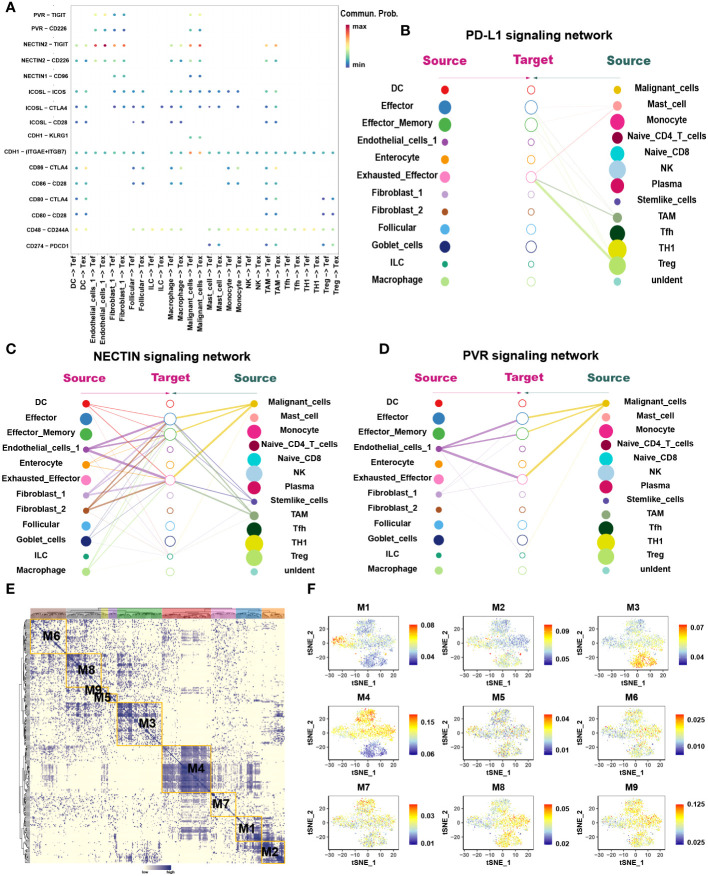
The interaction network and transcription regulatory network of CD8+ T cells. **(A)** Summary of selected ligand-receptor interactions between CD8+ T cells and TME-infiltrated cell types detected by CellChat. P-values are represented by the size of each circle. The color gradient indicates the level of interaction; blue and red colors correspond to the smallest and largest values respectively. **(B–D)** Hierarchical plot showing the inferred intercellular communication networks for PD-L1 **(B)**, NECTIN2 **(C)**, and PVR **(D)** signaling, respectively. The interactions are divided into sources and targets and were labeled by solid circle and hollow circle, respectively. The circle sizes in the hierarchical plot are proportional to the number of each cell type and the edge width represents the communication probability. **(E)** Heatmap of 9 identified regulon modules based on the regulon CSI matrix. **(F)** t-SNE map for all CD8+ T cells based on the regulon activity scores (RAS) of the respective regulon modules.

The CD8+ T cells were divided into nine sub-clusters and annotated into four cell types; naïve CD8+ T cells, effector memory CD8+ T cells (Tem), effector CD8+ T cells (Teff) and Tex (**Figure 2A**). To clarify the function of each cell type, we extracted the marker genes (**Table S1**) and loaded these into the Metascape (http://metascape.org/) (**Figure 2B, C**). The pseudotime trajectory revealed that CD8+ T cells became exhausted (**Figure 2D**), and inhibitory receptors (IRS) expression increased in a stepwise manner (**Table S2**). We clustered all the transcription factors surrounding the CD8+ T cells by single-cell regulatory network inference and clustering (SCENIC) analysis and divided them into nine modules using a clustering algorithm (**Figure 3E**; **Table S3**). Notably, Module 1 transcription factors including Nuclear receptor ROR-gamma (RORC), Nuclear receptor subfamily 1 group D member 1 (NR1D1), Peroxisome proliferator-activated receptor gamma (PPARG) and Sterol regulatory element-binding protein 2 (SREBF2) were significantly activated in Tex (**Figure 3F**).

#### 3.1.1 Loss of effector function during exhaustion of CD8+ T cells

The loss of Tex effector function is classed into three major categories: (1) upregulation of cell surface IRS, (2) inhibitory soluble factors and environmental factors such as interleukin10 (IL10), IL4, transforming growth factor-beta (TGF-β), and interferon alpha/beta (IFNα/β), and (3) immunosuppressive cells (11). We examined the immune checkpoints in different cell types (**Table S1**). Notably, IRS, including the inhibitory receptor T-cell immunoglobulin and mucin domain 3 (TIM3), lymphocyte activation gene 3 protein (LAG3), programmed cell death protein 1 (PDCD1), TIGIT, CD27, cytotoxic T-lymphocyte protein 4 (CTLA-4), and tumor necrosis factor receptor superfamily member 9 (TNFRSF9), were upregulated in Tex. Enrichment analysis showed that Teff was enriched in numerous proinflammatory pathways such as the IL-2, -3, -17, and -18 signaling pathways, whereas Tex was enriched in IL-4 and -10 immunosuppressive pathways and PD-1 signaling pathways (**Figures 2B, C**). GSVA analysis confirmed these results (**Figure 2G**). At the same time, the pseudotime analysis revealed that genes related to IRS were significantly upregulated along with the differentiation such as PD-1 and CTLA-4 (**Table S2**). The expression of immunosuppressive-related genes such as IL4, IL1RN, and IL4I1 were enhanced, whereas expression of immune activation-related genes such as IL18BP and IL5RA were reduced. This finding agrees with previous results where T cell exhaustion usually manifests as a stepwise loss of effector functions. CellChat analysis was undertaken to determine further the interaction between Tex and other cells in the TME. First, we analyzed the immunosuppressive receptors expressed by Tex, including TIGIT, CTLA-4, ICOS, and PDCD1, and found that different cells produced different ligand-receptor modes (**Figure 3A**). Endothelial and tumor cells mainly secreted poliovirus receptor (PVR) and NECTIN2, which acted on the TIGIT receptor on the surface of Tex. CD80 and CD86 secreted by DCs and TAMs interacted with CTLA-4. Regulatory T cells (Tregs) mainly secreted CD274 to act on PDCD1. Furthermore, analysis of the PD-L1 pathway regulatory network showed that Tregs were the main senders of PD-L1, with Tex being the main receivers (**Figure 3B**). Besides, cytokines such as PVR and NECTIN2 also participate in building the tumor immunosuppressive microenvironment. Analysis of the PVR pathway regulatory network showed that tumor and endothelial cells were the main senders, and immunosuppressive cells such as Tex were the receivers (**Figure 3D**). More interestingly, the fibroblast subgroup served as a mediator in this regulatory network, and this implies that this subgroup could be a potential target for new drugs. The NECTIN2 pathway regulatory network also showed multiple ligand-receptor modes; DCs, endothelial cells, fibroblasts, and TAMs were the main senders, and Tregs, Teff, and Tex were the main receivers (**Figure 3C**). Because PVR and NECTIN2 can both act on TIGIT, compared to the currently popular PDL1/PDLD1 blockers, TIGIT may not only reverse the exhaustion state of CD8 T cells but may also improve the tumor immunosuppressive microenvironment to a certain extent. Hence, we hypothesized that inhibition of TIGIT could be a new treatment for CRC. Our analyses showed that Tex play major roles in the immunosuppressive microenvironment, and the depletion of CD8+ T cells is an inevitable outcome in TME.

#### 3.1.2 Metabolic remodeling in the CD8+ T cell exhaustion process

We constructed the differentiation trajectory of CD8+ T cells using pseudotime analysis, in which effector memory CD8+ T cells were present at the initial location of the differentiation trajectory, gradually differentiated into Teff and finally convert into Tex which located at the end of the differentiation trajectory (**Figure 2D**). As we all know, under chronic inflammation such as during cancer, autoimmunity, and chronic infections, Teff transform into Tex (23). Thus, we identified the DEGs (**Table S1**) among the Teff and Tex and performed enrichment analysis (**Figures 2B, C**). It was found that the metabolic patterns of Teff and Tex were significantly different. The glucose metabolic process was enriched in Teff while lipid metabolism processes such as lipid biosynthesis and the cholesterol metabolic pathway were highly enriched in Tex. GSVA also support this finding (**Figure 2G**). We extracted genes whose expression increased in the differentiation trajectory and then conducted enrichment analysis. The pathways such as fatty acid biosynthesis and omega-3, -6, and -9 fatty acids (FAs) synthesis were all enriched (**Figure 2E**). These results implied that the differentiation of T cells was related to lipid metabolism remodeling, and abnormal lipid accumulation may be the energy source for Tex. The DEGs analysis, Pseudotime analysis and GSVA all showed that the PPARG pathway was highly expressed in Tex. A previous study showed activation of the PPAR pathway in the metabolic regulation of lipid and lipoprotein levels (24). Based on these results, we suspect that the lipid metabolism remodeling in Tex is attributed to the activation of the PPARG pathway. To verify this hypothesis, we performed SCENIC analysis to reveal the abnormal transcriptional regulatory network of Tex. Without suspension, PPARG was significantly enriched in Tex. This further demonstrates that the PPARG transcription factor may play an important role in lipid reprogramming in Tex. In addition, we also enriched the RORC, NR1D1, and SREBF2 transcription factors in the M1 module, which are also closely associated with lipid metabolism (25–27). Our results suggested that transcription factors (TFs) such as PPARG and SREBF2 may participate in the metabolic remodeling in Tex and act as latent targets to reverse this process.

### 3.2 Myeloid cells

**Figure 4:**
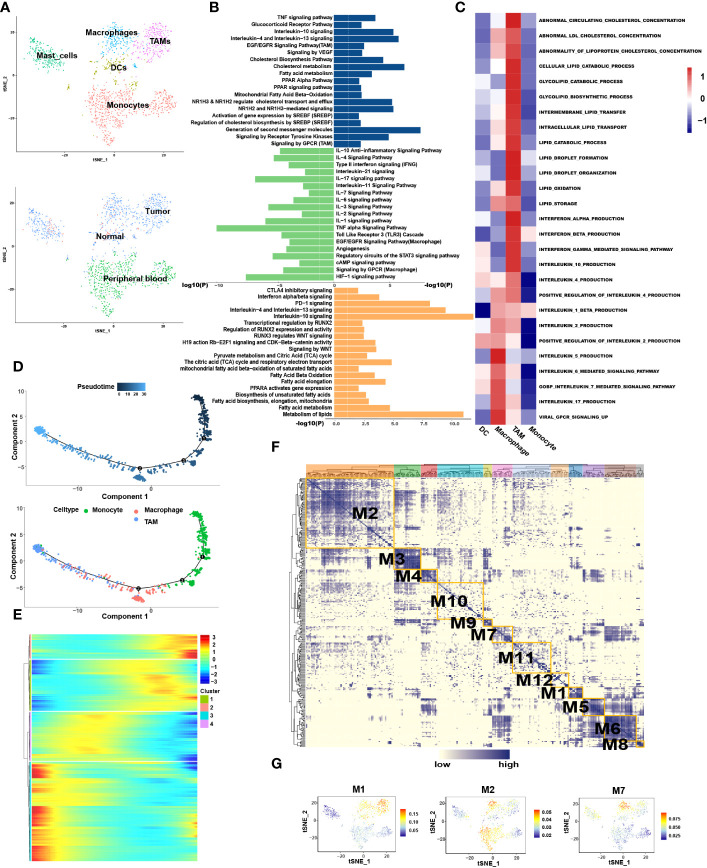
Macrophages resulted from M1 polarization, whereas TAMs resulted from M2 polarization in CRC. **(A)** tSNE plots showing 4 sub-cell types of CD8+ T cells (upper) and their tissue origins (lower). **(B)** GO and KEGG pathway enrichment analyses of marker genes of TAMs (blue color), Teff (green color) and DCs (orange color). The height of each barplot shows the log10 of P-value calculated using the Metascape database. **(C)** The heatmap illustrates the activity of biological process and signaling pathway in each cell type by GSVA. **(D)** Differentiation trajectory of monocytes, macrophages and TAMs in CRC, color-coded for pseudotime (upper) and sub-cell types (lower). **(E)** Pseudo-heatmap of genes altered in the differentiation process of monocytes, macrophages, and TAMs in CRC, grouped into four clusters. **(F)** Heatmap of 12 identified regulon modules based on the regulon CSI matrix. **(G)** Selected regulon models which upregulated in TAMs (M1) and Macrophages (M2, M7) showed in t-SNE map for myeloid cells.

Myeloid cells are abundant critical components of the TME which are heterogeneous mixture of cell types having both tumor stimulating and suppressing activities. Analysis of the myeloid cells revealed five distinct sub-clusters: monocytes, macrophages, TAMs, DCs, and mast cells (**Figure 4A**). Among them, macrophages and TAMs can be activated and polarized into M1 (classically activated) and M2 (selectively activated) phenotypes under the influence of external conditions and stimulus factors. M1 cells usually show pro-inflammatory activity, while M2 cells exhibit tumor-promoting phenotypes characterized by high levels of immunosuppressive markers and anti-inflammatory factors (28).

Interestingly, when traced back to the tissue source, monocytes were present primarily in the blood, while macrophages and TAMs occurred in most tumor tissues (**Figure 4A**). Pseudotime analysis showed that monocytes differentiated into macrophages when they entered the TME from the blood and finally differentiated into TAMs (**Figure 4D**).

#### 3.2.1 TAMs are engaged in constructing the immunosuppressive microenvironment

Enrichment analysis revealed that Pathways associated with proinflammation were enriched in macrophages, such as IL-1, -2, -3, -11, -17, -18, -21, TNF alpha and interferon alpha/beta signaling pathways while macrophages also exhibit few M2-like function such as IL-4, -10, and TGF-beta receptor signaling (**Figure 4B**). In contrast, pathways associated with tumor promotion and immunosuppression were enriched in TAMs such as arachidonic acid metabolism, matrix metalloproteinase (MMP), the vascular endothelial-derived growth factor (VEGF), IL-4, -10, -13 and PD-1 signaling. Few M1-like functional pathways were also present in TAMs such as interferon gamma and TNF signaling pathways. GSVA analysis also disclosed that IFN alpha/beta signaling was enriched in TAMs, while IL-5, -6, -7 and -17 were enriched in macrophages (**Figure 4C**). In conclusion, macrophages and TAMs exhibit mixed M1 and M2 phenotypes among which macrophages mainly exhibit M1 phenotype, whereas TAMs mainly exhibit M2 phenotype. Combined above results with tissue origination and pseudotime analysis, we speculated that once monocytes from the peripheral blood entered the tumor tissues, they initially differentiated into M1-type macrophages and finally differentiated into M2-type TAMs, alongside enhanced immunosuppressive effects.

SCENIC analysis was performed to determine the changes in TFs during the transformation of macrophages into TAMs (**Figures 4F, G**). We found that STAT4, NFκB1, NFκB2 and RUNX1 were enriched in macrophages (**Table S5**) in which STAT4 has been proved to mediates the JAK-STAT-related pathways and participates in the conduction of the IL-12, -21, -23 and -35 signaling pathways (29). NFκB1 and NFκB2 can promote the polarization of macrophages to M1 type (30). Conversely, MAF, ETV5 and EGR2 were highly expressed in TAMs in which MAF regulates the activation of IL-4 pathway and ETV5 is related to blood vessel growth and activation of the IL-10 pathway (31–33). The expression of EGR2 was found to be related to the activation of the IL-4 and TGF-β functional pathways (34, 35).

**Figure 5:**
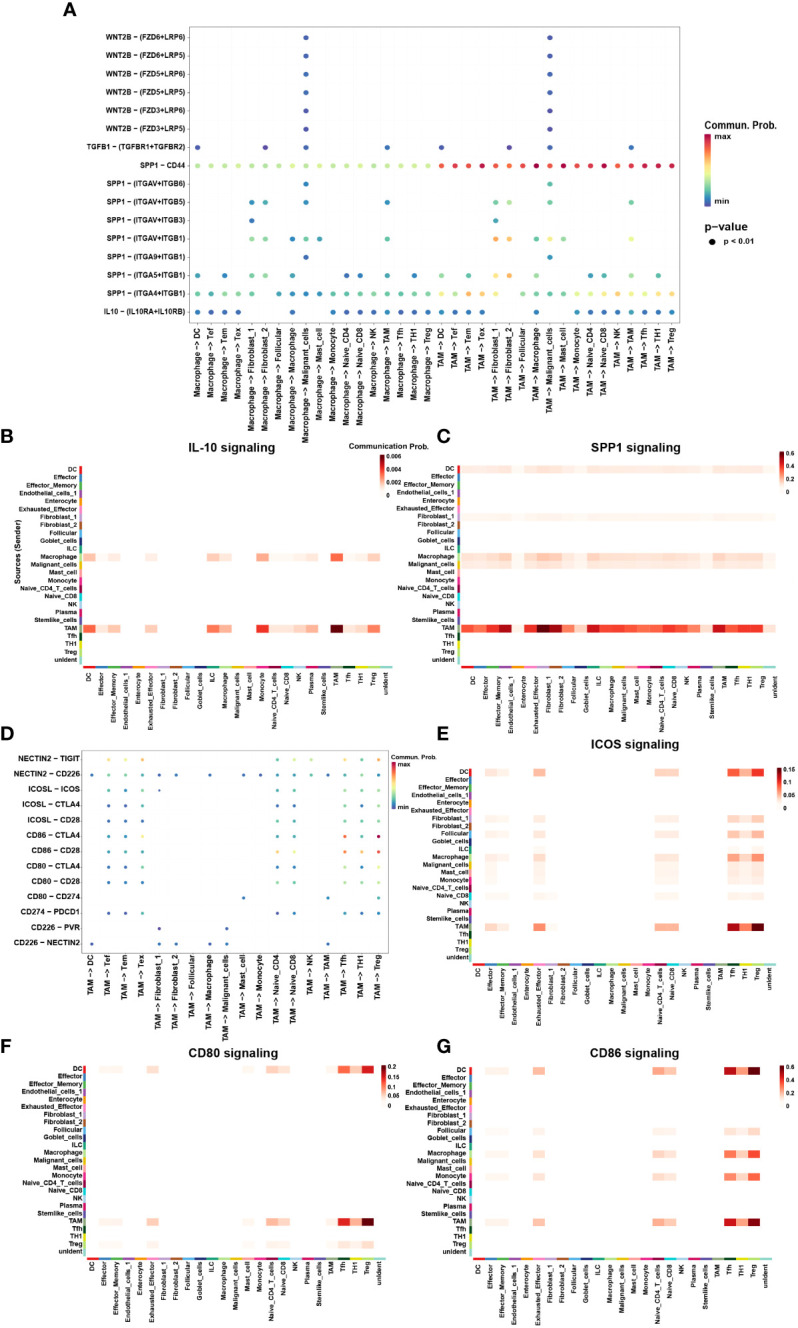
The interaction network of macrophages and TAMs. **(A)** Summary of selected soluble factor-receptor interactions among macrophages, TAMs and TME-infiltrated cell types. **(G)** Summary of selected immune checkpoints-receptor interactions between TAMs and TME-infiltrated cell clusters. P-values are represented by the size of each circle. The color gradient indicates the level of interaction; blue and red colors correspond to the smallest and largest values respectively. **(B, C, E–G)** The heatmap plot showed the inferred intercellular communication network for SPP1 **(B)** and IL-10 **(C)**, CD80 **(E)**, CD86 **(F)**, and ICOSL **(G)** signaling of myeloid cells and TME-infiltrated cell clusters, respectively. The interactions are divided into sources (labeled on y-axis) and targets (labeled on x-axis). The color gradient represents the communication probability; white and red colors correspond to the smallest and largest values respectively.

Finally, we utilized CellChat to investigate the interactions between TAMs and other cell subtypes in TME (**Figure 5D**). Compared to macrophages, TAMs participated more in constructing the immunosuppressive microenvironment. The immunosuppressive ligands secreted by TAMs, such as CD80, CD86, CD274, ICOSL and NECTIN2 showed evident interactions with other receptors such as CTLA-4, PD-1, ICOS and TIGIT expressed by other cells, especially T cells (**Figure 5E–G**). In addition to IRS, TAMs also secreted immunosuppressive soluble cytokines such as IL-10 and SPP1 (**Figure 5A**). Interestingly, TAMs were the main secretors of IL-10, whereas macrophages were the main receivers of IL-10 (**Figure 5B**). This suggested a possible positive feedback loop between macrophages and TAMs. Once macrophages had differentiated into TAMs, TAMs possibly secrete IL-10 acting on macrophages to promote the differentiation process (**Figure 5B**). TAMs also secreted SPP1 which have been found mediating macrophage polarization and facilitates immune escape in lung adenocarcinoma (36). SPP1 secreted by TAMs could interact with almost all cells in TME, including DCs, Tregs, Tex, fibroblasts, and malignant cells (**Figure 5C**). Interestingly, TAMs were not only the main secretors of SPP1 but also the main receivers. It may be attributable to the M2 phenotype of TAMs in this study.

#### 3.2.2 Lipid metabolism reprogramming in TAMs

Lipid metabolism associated genes such as PPARA were highly expressed in the TAMs. In order to explore whether there was lipid metabolism remodeling in TAMs similar to that in T cell exhaustion, marker genes of macrophages and TAMs were extracted for functional enrichment analysis. The results showed that, compared to macrophages, TAMs are enriched in more lipid metabolic pathways such as cholesterol biosynthesis and fatty acid metabolism, such as the “PPAR Alpha Pathway”. “Regulation of cholesterol biosynthesis by sterol regulatory-element binding proteins (SREBP)” and “ Oxysterols receptor LXR-beta (NR1H2) and Oxysterols receptor LXR-alpha (NR1H3) Mediated signaling “(**Figure 4B**). GSVA also further confirmed enhanced synthesis of cholesterol and lipid droplets in TAMs (**Figure 4C**). Pseudotime analysis showed that the expression of genes related to lipid output, such as ABCA1 and ABCG1, was gradually enhanced during macrophage differentiation (**Table S4**). It is reasonable to speculate that the differentiation of macrophages into TAMs is accompanied by lipid metabolism remodeling.

Among the functional pathways enriched in TAMs, three transcription factors attracted our attention, namely SREBF, NR1H2 and NR1H3. Further SCENIC analysis reported the abnormal transcriptional regulatory network in TAMs (**Table S5**). As shown in **Figure 4G**, transcription factors in module 1 were significantly activated in TAMs, which include the SREBF and NR1H3. Among them, SREBF functions in the transcriptional regulation of genes involved in the biosynthesis and uptake of lipids, promoting fatty acid synthesis and inducing M2 phenotype of TAMs (37, 38). NR1H2 and NR1H3 act as transcription factors engaged in lipid metabolism synthesis and are important modulators of the SREBP-1c pathway at the transcription level, where they regulate gene expression linked to cholesterol transport and efflux in hepatic lipogenic cells (39). We were particularly interested in the cholesterol efflux function mediated by NR1H2 and NR1H3. Increased cholesterol outflow increased lipid content in the TME to provide nutrition for tumor cell growth and destroyed the lipid raft of TAMs to weaken the Toll-like Receptor 4 (TLR4) signaling pathway (39). It also enhanced the IL-4 pathway, weakened the interferon pathway (40), and has an unexpected role in the polarization of TAMs to M2. We speculated that reprogramming of lipid metabolism in TAMs is involved in the remodeling of immune functions, to a certain extent.

Therefore, SREBF and NR1H3 play important roles in lipid metabolism reprogramming in TAMs. TAMs and Tex have both undergone lipid metabolism remodeling, reflecting the important role of lipid metabolism in the process of T cell exhaustion and TAMs polarization to M2 type. However, there are significant differences between these two kinds of cells, which are mainly manifested in the differences in the transcription regulatory factors. Hence, we suspect that SREBF and NR1H3 may be important targets to prevent or reverse the polarization from TAMs to M2.

#### 3.2.3 DCs exhibit a similar pattern to TAMs in metabolism remodeling and construction of the immunosuppressive microenvironment

DCs are the most potent antigen-presenting cells in the immune system and are central players in the adaptive immune response. DEGs analysis revealed that DCs exhibited highly expressed immunosuppressive cytokines, such as IL-4, -10, and IFNα/β (**Table S1**). Further enrichment analysis showed that IL-4, -10, and -13, interferon alpha/beta, PD-1, and CTLA-4 inhibitory signaling pathways were enriched in DCs (**Figure 4B**). CellChat analysis found DCs exhibited a similar pattern to TAMs in secreting immunosuppressive cytokines, especially ICSO (**Figure 5E**), CD80 (**Figure 5F**) and CD86 (**Figure 5G**). These heatmaps indicated that DCs and TAMs were the main secretors participating in the exhaustion process of CD8+ T cells, synergistically promoting the construction of the immunosuppressive microenvironment.

At the same time, enrichment analysis showed that lipid metabolism, fatty acid metabolism, and PPARA signaling pathways were highly enriched in DCs (**Figure 4B**). Except for aberrant lipid storage, the PPARs pathway also enhances TCA cycle, resulting in citric acid accumulation. These conditions provide the substrate for the *de novo* synthesis of fatty acids and intracellular lipid droplets. Other pathways were also enriched, including Wnt signaling and CDK-beta-catenin activity. Wnt5 has been proved to act on Frizzled (FZD) family receptors on DCs and trigger the activation of downstream PPAR pathways through activation of β-catenin signals to remodel lipid metabolism in melanoma (41) (**Figure 4B**). Transcriptional regulation by RUNX2 and RUNX3, regulating Wnt signaling was enriched in DCs. SCENIC analysis demonstrated that RUNX2 was highly expressed in DCs (**Figure S3D**). These results implied that lipid metabolism remodeling in DCs might also depend on the core Wnt/β-catenin/PPAR signaling pathway regulated by the RUNX family.

Based on the above analysis, we speculate that lipid metabolism reprogramming in DCs is involved in reconstructing the immunosuppressive microenvironment.

### 3.3 Fibroblast cells

**Figure 6:**
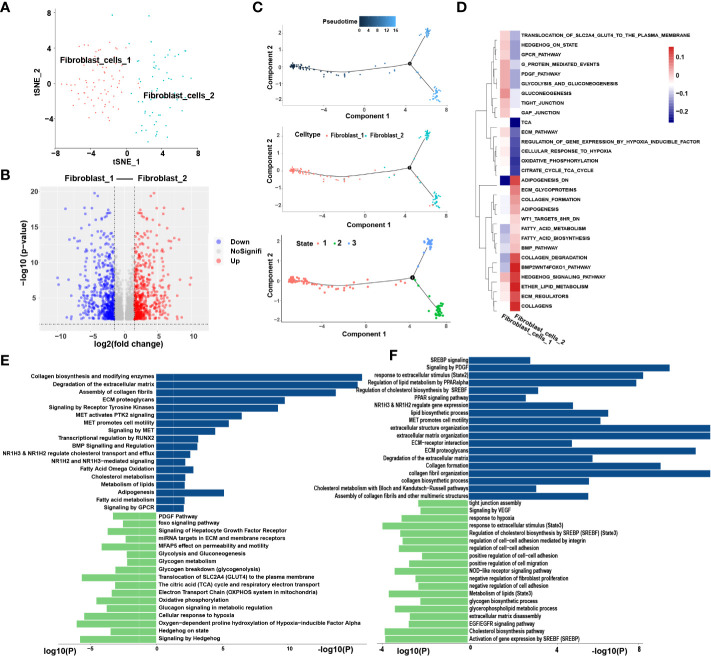
Similar metabolic and functional remodeling in fibroblasts. **(A)** tSNE plots showing 2 sub-cell types of fibroblasts. **(B)** The volcano plot illustrated the DEGs of each sub cluster, statistically significant DEGs were defined with p< 0.05 and [logFC] > 1 as the cut-off criterion. **(C)** Differentiation trajectories of fibroblasts color-coded for pseudotime (upper) and sub-cell types (median) and states (lower). **(D)** The heatmap illustrates the activity of biological process and signaling pathway in each cell type by GSVA. **(E)** GO and KEGG pathway enrichment analyses of marker genes of fibroblast-2 (blue) and fibroblast-1 (green). **(F)** Enrichment analyses of marker genes of state2 (blue) and state3 (green) cluster. The height of each barplot shows the log10 of P-value calculated using the Metascape database.

We extracted 145 fibroblast cells classified into two clusters: fibroblast-1 and fibroblast-2 (**Figures 6A, B**). Pseudotime analysis revealed that fibroblast-1 was present at the initial stage of the differentiation trajectory, and fibroblast-2 was present at the end. Interestingly, fibroblast_2 also differentiated into two distinct subtypes, state2 and state3 (**Figure 6C**).

#### 3.3.1 Similar metabolic and functional remodeling in fibroblasts

Enrichment analysis showed that compared to fibroblast-1, fibroblast-2 is more involved in extracellular matrix (ECM) degradation and promotion of cell motility regulated by MET signaling pathway (**Figure 6D**). Interestingly, the metabolic patterns between the two clusters are totally different. The pathways related to lipid cholesterol and fatty acid metabolism were significantly enriched in fibroblast-2. In contrast, fibroblast-1 exhibited carbohydrate metabolism pattern (**Figure 6E**). Furthermore, the two subgroups of fibroblast-2 both exhibited patterns of ECM regulation and lipid metabolism, while the state2 subgroup showed stronger patterns of lipid metabolism remodeling, ECM degradation and promotion of cell motility regulated by MET signaling pathway compared to state3 (**Figure 6F**). Among these, several pathways highly enriched in fibroblast-2 aroused our attention, such as”NR1H2&NR1H3 regulate gene expression linked to cholesterol transport and efflux”, “NR1H2 and NR1H3 Mediated signaling” and “transcriptional regulation by RUNX2”. SCENIC analysis also showed that NR1H2, NR1H3and RUNX were upregulated in fibroblast-2 (**Figure S3D**). It was highly consistent with that of TAMs. Above results revealed that enhanced lipid metabolism and abnormal lipid accumulation may also occur in the differentiation from fibroblast-1 to fibroblast-2.

### 3.4 The infiltration of tumor-educated immune cells is associated with a worse prognosis in CRC

We performed digital cytometry analyses using CIBERSORTx to evaluate the abundance of tumor stromal and immune cell subsets analyzed previously in patients from The Cancer Genome Atlas-Colon Adenocarcinoma (TCGA-COAD) data. We established a new risk model using stepwise regression to evaluate the association between cell fractions and prognostic outcomes and identify the optimal coefficient for each subgroup in the training cohort. Finally, we selected sixteen subgroups to construct the model. The formula for the risk model is as follows:

Riskscore=-2.373*Fibroblast_cells.0+9.172*Fibroblast_cells.1+6.570*Myeloid.cell.0+5.484*Myeloid.cell.1-827.566*Myeloid.cell.2+9.532*Myeloid.cell.5+18.344*Myeloid.cell.6-.645*Myeloid.cell.7+16.412*CD8_T_cells.0-0.766*CD8_T_cells.1+7.595*CD8_T_cells.2+40.164*CD8_T_cells.3+7.233*CD8_T_cells.4-28.620*CD8_T_cells.5+8.862*CD8_T_cells.7+4.852*CD8_T_cells.8 (The correspondence between each subgroup and sub cell type was applied in **Supplementary Table 9**).

**Figure 7:**
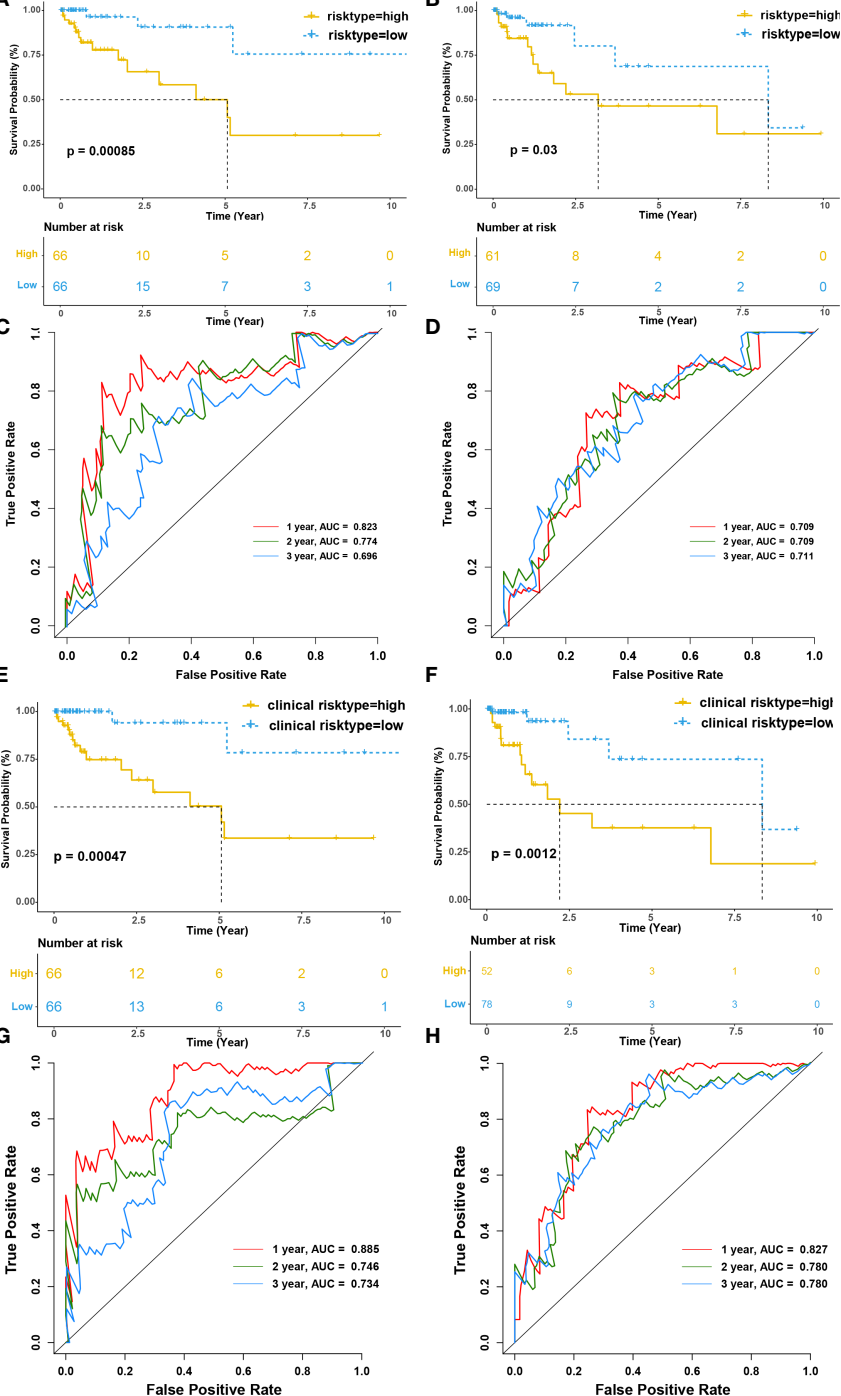
Infiltration of tumor-educated immune cells is associated with a worse prognosis in CRC. **(A, B)** Kaplan–Meier survival curves of immune risk model for the training **(A)** and testing cohorts**(B)**, respectively. **(C, D)** The time-dependent ROC curves of immune risk model for 1-, 2- and 3- OS year in the training **(C)** and testing cohorts **(D)**, respectively. The areas under the ROC curve for 1-, 2- and 3- year OS were 0.823, 0.774, and 0.696 in the training cohort and 0.709, 0.709 and 0.711 for 1-, 2- and 3- year OS in the testing cohort. **(E, F)** Kaplan–Meier survival curves of clinical risk model for the training **(E)** and testing cohorts**(F)**, respectively. **(G, H)** The time-dependent ROC curves of clinical risk model for 1-, 2- and 3- OS year in the training **(G)** and testing cohorts **(H)**, respectively. The areas under the ROC curve for 1-, 2- and 3- year OS were 0.823, 0.774, and 0.696 in the training cohort and 0.709, 0.709 and 0.711 for 1-, 2- and 3- year OS in the testing cohort.


Then, we evaluated the prognostic value of the risk model for overall survival (OS). Patients in the high-risk group had a significantly worse OS than the low-risk group both in training and testing cohort (p<0.001 and p=0.03, respectively) (**Figures 7A, B**). The model’s accuracy was verified using time-dependent receiver operating characteristic (ROC) curves, which confirmed the reliability of the prognoses for both cohorts. The area under the ROC curve for the risk score was 0.823, 0.774, and 0.696 for 1-, 2- and 3-year OS in the training cohort, versus 0.709, 0.709, 0.711 in the testing cohort. (**Figures 7C, D**). Furthermore, we used a stepwise multivariate Cox regression to construct a new clinical model incorporating riskscore, TNM stage, gender, and age in the training cohort and selected riskscore and TNM stage to construct the model. The formula for the clinical risk model is as follows:

Clinical riskscore=0.472*riskscore+0.582*stage

Interestingly, riskscore and TNM stage were both independent prognosis factors (p<0.001 and p=0.038, respectively). The patients were separated into two subgroups according to the median clinical riskscore. KM survival analysis revealed that high clinical risktype had a significantly worse OS than low clinical risktype both in training cohort and testing cohort (p<0.001 and p=0.0012, respectively) (**Figures 7E, F**). The areas under the ROC curve for 1-, 2-, and 3-year OS was 0.885, 0.746, and 0.734 for 1-, 2- and 3-year OS in the training cohort, versus 0.827, 0.780, 0.780 in the testing cohort. (**Figures 7G, H**), which was better than the immune risk model.

We also applied other immune risk model that have been reported and TNM stage for validation. Patients in the high-risk/high-stage (III-IV) group showed a significantly worse OS than the low-risk/low-stage (I-II) group (p<0.001 and p=0.015, respectively) (**Figures S6B, S6A**). The area under the ROC curve for the risk score was 0.758, 0.760, and 0.717 for 1-, 2- and 3-year OS for the immune risk model, versus 0.726, 0.636, 0.650 for the TNM stage model (**Figures S6D, S6C**).

## 4 Discussion

Currently, the treatment of CRC, especially advanced CRC, still remains challenging. Although ICB has made some progress, only a small number of people benefit from it due to low efficiency, high drug resistance, severe toxicity and potential for relapse. A recent study found that both tumor cells and tumor-infiltrating cells are involved in the development of drug resistance (42). As for the other defects are due to insufficient of systematic cognization of immunotherapy. Recent studies related the heterogeneity identified by scRNA-seq in human cancers to cell types found in murine tumor models and identified many functional sub clusters responsible for the poor immunotherapy response such as CXCL13+BHLHE40+ Th1-like cell population (43), C1QC+SPP1+TAMs (13), XCR1+CADM1+cDC, CD1A+ CD172A+cDC (44), which provides many valuable insights for the development of clinical strategies. Although recent studies have made significant progress in resolving the problem of heterogeneity, there are obvious shortcomings in elucidating the common features of newly defined immunosuppressive cells such as Tex and TAMs.

In this study, we leverage the advantage of integrated scRNA-seq and bulk RNA-seq as well as a variety of bioinformatics analyses to clarify the heterogeneity and convergence of TME in CRC. Eight main cell types were identified preliminarily and 25 sub cell types were further distinguished after improving the resolution. It was found that the metabolic patterns and immunophenotypes displayed by each cell type were extremely different. However, we were surprised to find that multiple sub cell types manifest similar metabolic patterns and immunosuppressive functions at the end of differentiation trajectory. Meanwhile, we found similar immunosuppressant ligand-receptor pairs in Tex, TAMs, and fibroblast-2 sub cell types by intercellular communication network analysis, and similar TFs regulating lipid metabolic remodeling were found in transcription factor regulatory network analysis.

Since it is impossible to adequately characterize the tumor microenvironment in CRC, we selected several specific cell types, such as CD8+ T cells, myeloid cells and fibroblasts representing the main components of the TME, to illustrate its heterogeneity and convergence. Our key conclusions are as follows:

First, we identified that the immunosuppressive microenvironment of CRC was co-shaped by immune cells, stromal cells and tumor cells. Meanwhile, for each cell type the cells closer to the end of their differentiation trajectory showed more immunosuppressive characteristics, such as exhaustion in CD8+ T cells and polarization to the M2 phenotype in TAMs. In this process, proinflammatory functions were inhibited, whereas immunosuppression functions were enhanced. In addition, the intercellular communication network showed more active secretion of immunosuppressive cytokines by cells closer to the end of their differentiation trajectory. For example, in the regulation of IRS, exhaustion was the inevitable outcome of CD8+ T cells mediated by various cells in the TME. At the same time, different cells manifested different ligand modes. Tumor cells mainly secreted PVR and NECTIN2 to act on the TIGIT receptor. CD80 and CD86 secreted by DCs and TAMs interacted with CTLA-4 and Tregs mainly secreted CD274 to act on PDCD1. Soluble cytokines such as IL-10 and SPP1 were secreted by TAMs. More importantly, there are multiple positive feedback loops among intercellular subgroups. For example, the network analysis of IL-10 implied a potential positive feedback loop between macrophages and TAMs to promote the differentiation process. The positive feedback loop may equally be applied to SPP1 in TAMs to maintain the M2 phenotype. Therefore, we speculate that these inhibitory ligand-receptor pairs and positive feedback loops of cytokines are involved in the construction and maintenance of the immunosuppressive microenvironment, and are also important potential targets for our immunotherapy and targeted therapy.

As mentioned above, we mapped the differentiation pathways of each cell type and found that different subgroups of each cell type had different metabolic patterns. Interestingly, although the metabolic patterns of each subgroups within the certain cell types were diverse, those cells close to their terminal differentiation trajectory showed similar metabolic patterns, namely enhanced lipid metabolism and abnormal accumulation of intracellular lipid. SCENIC analysis revealed that the transcription factors that regulate lipid metabolism remodeling in each cell type partially overlapped. The most representative transcription factors are PPARG, SREBF, NR1H2, and NR1H3. The genes regulated by NR1H2 and NR1H3 were linked to cholesterol transport and efflux, and the outflow of cholesterol could destroy the lipid rafts on cell membranes, attenuating the TLR4 signaling pathway. Furthermore, increased cholesterol outflow also enhanced the IL-4 pathway and attenuated the IFN pathway. This phenomenon implied that enhanced intracellular lipid metabolism might be an important factor in the transformation of immune function, and transcription factors involved in lipid metabolism remodeling in cells may be potential therapeutic targets to reverse immunosuppression.

We applied CIBERSORTx algorithm to quantitatively assess the association between the proportion of cell subgroups in TME and prognosis in CRC. KM survival analysis and ROC curve analysis suggest that our immune risk model is an effective clinical prediction tool, which can improve the accuracy of survival prediction in CRC patients. Furthermore, the clinical risk model constructed by incorporating immune risk type and TNM stage could not only predict the survival prognosis of colorectal cancer patients, but also had significantly better AUC values at 1, 2 and 3 years than immune risk model both in training and testing cohorts. In addition, validation prognostic model showed similar prognostic value to our immune risk model whereas worse than our clinical risk model. This indicates that the risk prognosis model based on cell proportion in TME can supplement the existing clinical prognosis criteria and is a method with great prospects in clinical practice applications.

Currently, conventional RNA sequencing is the mainstream sequencing technology, but its gene expression level is the mixed expression of all cells in the tissue after lysis. Although simple and intuitive, it cannot reflect the gene expression of a single cell or a single cell group. With the further analysis, the accuracy of sequencing is required to be higher and higher. With its high-precision sequencing analysis, scRNA-seq has become a powerful technology in modern medical research, but this technology cannot be applied to most preserved tissue samples and is expensive, so it cannot be used as a routine clinical treatment project. The deconvolution algorithm CIBERSORTx can not only deconstruct ordinary RNA-seq to achieve the secondary utilization of data, but also to some extent make up for the shortcomings of scNA-SEQ tissue samples, such as high requirements, high price and insufficient sample size. More importantly, with the progress of sequencing technology, the cost of ordinary RNA-SEQ will gradually decrease, while the accuracy and data volume of scRNA-seq will continue to improve. Meanwhile, deconvolution algorithms like CIBERSORTx will also continue to improve, which means that in the near future, more and more patients with colorectal cancer can benefit from the high-precision analysis of scRNA-seq while enjoying the low cost of ordinary RNA-seq.

Although the heterogeneity and convergence of CRC microenvironment were further analyzed by using scRNA-seq and constructed an immune risk prognostic model based on CIBERSORTx algorithm and bulk RNA-seq data in this study, there are still some limitations. First of all, our data sources are all public databases, so we cannot obtain all clinical information that is meaningful for the study, such as tumor size, location, differentiation degree, pathological classification, immunohistochemical results, surgical methods, postoperative radiotherapy and chemotherapy, and patients’ underlying diseases, etc. This will inevitably lead to the introduction of confounding factors in the construction of the prognostic model, and cause certain deviations in the final results. Secondly, although our single-celled sequencing analysis at the cellular level to reveal the gene expression, and through a variety of biological information analysis method to predict and infer the trajectory, regulation and control of transcription factors, cell differentiation and intercellular communication network, but has not been experimental verification, the follow-up still need further perfect the related experiments *in vivo* and *in vitro* in order to strengthen the reliability of conclusions.

## 9 Conclusion

This study further revealed the heterogeneity and convergence in TME, especially the high consistent lipid metabolism remodeling and immunosuppressive phenotype during the differentiation of each cell subpopulation, providing a new perspective for the targeted therapy and immunotherapy of colorectal cancer. Meanwhile, CIBERSORTx algorithm was used to integrate scRNA-seq and bulk RNA-seq data to construct immune risk model and clinical risk model, providing reference value for prognostic analysis of colorectal cancer patients. In conclusion, this study provides a new perspective for understanding the heterogeneity and convergence of the TME and will aid the development of immunotherapies to treat CRC.

## Data availability statement

Publicly available datasets were analyzed in this study. This data can be found here: Gene Expression Omnibus (GEO, http://www.ncbi.nlm.nih.gov/geo/) database, GSE146771.

## Author contributions

JY and JM designed the study and reviewed the literature. SX performed the data analyses and wrote the manuscript for the study. YC and DC downloaded the data from GEO database and participated in the drafting of the manuscript. WC contributed to the reviewing of the literature. YX contributed to the literature research. All authors contributed to the article and approved the submitted version.

## Glossary

**Table:**

CRC	colorectal cancer
SCENIC	single-cell regulatory network inference and clustering
TCGA	The Cancer Genome Atlas
HER2	human epidermal growth factor receptor 2
EGFR	epidermal growth factor receptor
TME	tumor microenvironment
Tex	exhausted CD8 T cell
TAM	tumor-associated macrophages
CAF	cancer-associated fibroblasts
ICB	Immune checkpoint blockade
PD-1	programmed cell death 1
CTLA-4	cytotoxic T lymphocyte-associated protein 4
dMMR	mismatch-repair-deficient
MSI-H	microsatellite instability-high
MSI	microsatellite instability
IRS	inhibitory receptors
scRNA-seq	single-cell RNA sequencing
TNM	the tumor
nodes	and metastasis
QC	quality control
ILC	innate lymphoid cell
DC	dendritic cells
BEAM	branch expression analysis modeling
Teff	T effector cell
DEG	differentially expressed genes
AMPK	Adenosine 5&rsquo;-monophosphate -activated protein kinase
GSVA	gene set variation analysis
FA	fatty acid
SREBP	Sterol regulatory element binding protein
FASN	atty acid synthase
ACC	Acetyl-CoA Carboxylase
HMG-CoA	3-hydroxy-3methylglutary-coenzyme A
ACLY	ATP-citrate lyase
PPAR	peroxisome proliferator-activated receptor
TF	transcription factors
TGF-&beta;	transforming growth factor-beta
IFNα/β	interferons alpha and beta
TNF	tumor necrosis factor
IL	interleukin
Treg	regulatory T cells
PVR	poliovirus receptor
TIGIT	T cell immunoreceptor with immunoglobulin and ITIM domain
ICOSL/ICOS	Inducible Co-Stimulator Ligand/Inducible Co-Stimulator
TIM3	the inhibitory receptor T-cell immunoglobulin and mucin domain 3
LAG3	lymphocyte activation gene 3 protein
MSI-L	microsatellite instability-low
VEGF	vascular endothelial-derived growth factor
ZEB1	zinc finger E-box binding homeobox 1
MMP	matrix metalloproteinase
TLR4	Toll-like Receptor 4
TCA	tricarboxylic acid
FZD	Frizzled
GPCR	G-protein-coupled receptor
BMP	bone morphogenetic protein
Dvl	Disheveled
AhR	aryl hydrocarbon receptor
EMT	epithelial&ndash;mesenchymal transition
TCGA-COAD	The Cancer Genome Atlas-Colon Adenocarcinoma
OS	overall survival
ROC	receiver operating characteristic
KM	Kaplan-Meier
GEO	Gene Expression Omnibus
PC	principal component
tSNE	t-distributed stochastic neighbor-embedding
CSI	Connection Specificity Index
TPM	transcripts per million
NR1H3	Liver X receptors alpha
NR1H2	Liver X receptors beta
