# Supplementary material for: Single-cell transcriptome analysis reveals heterogeneity and convergence of the tumor microenvironment in colorectal cancer
Source: Front Immunol. 2023 Jan 4;13:1003419. doi: 10.3389/fimmu.2022.1003419 (PMC9845924; doi:10.3389/fimmu.2022.1003419)
Supplement: Supplementary file 1 [file DataSheet_1.zip › Supple-Legend.docx]

**Supplementary Figure 1:**

A-C). top 3000 variance genes were included for further PCA analysis. D). Dot plot revealed top 20 dimensions for further analysis. E). Interaction network dominated by CD8+ T cells、Myeloid cells、Fibroblasts and Epithelial cells. F) heatmap manifests top five marker genes of 8 major cell types identified in this profile.

**Supplementary Figure 2:**

A) tSNE plot of four sub groups of CD8+ T cells. B) the origination of tissues (tumor, adjacent tumor or blood) C) bubble plot manifests top five marker genes of each sub groups. D) top ten transcription factors of Tex、cluster 5 and cluster 6.

**Supplementary Figure 3:**

A-B) top 10 transcription factors of TAMs and Macrophages. C) Transcription factors as MAF、ETV5、EGR2 and STAT4 expression in all single cells of CD8+ T cells. D) top 10 transcription factors of DCs、Fibroblast-1 and Fibroblast-2.

**Supplementary Figure 4:**

A) tSNE plot of four 9 groups of Myeloid cells. B) the origination of tissues (tumor, adjacent tumor or blood). C) Heatmap of identified regulon modules based on regulon connection specificity index (CSI) matrix. D) t-SNE map for all Myeloid cells based on regulon activity scores (RAS) of regulon module.

**Supplementary Figure 5:**

A) tSNE plot of four 9 groups of Epithelial cells (upper) and the origination of tissues (lower).

B) The volcano plot illustrated the DEGs of malignant cells (red) and monocytes (blue)., statistically significant DEGs were defined with p < 0.05 and [logFC] > 1 as the cut-off criterion.

C) Enrichment analyses of marker genes of state2 (blue) and state3 (green) cluster. The height of each barplot shows the log10 of P-value calculated using the Metascape database. D) The heatmap illustrates the activity of biological process and signaling pathway in each cell type of epithelial cells by GSVA.

**Supplementary Figure 6:**

(A–B) Kaplan–Meier survival curves of TNM stage (A) and immune risk model(B), respectively. (C–D) The time-dependent ROC curves of immune risk model for 1-, 2- and 3- OS year in the TNM stage (C) and immune risk model (D), respectively. The areas under the ROC curve for 1-, 2- and 3- year OS were 0.726, 0.636, and 0.650 of the TNM stage and 0.758, 0.760 and 0.717 for 1-, 2- and 3- year OS of immune risk model.

**Supplementary Figure 7:**

(A-E) mRNA expression of FASN, CPT1A, SCD, CD36, FABP in fibroblasts (NF and CAF) are shown as relative fold to control normalized to β-actin. (F-K) mRNA expression of FASN, CPT1A, SCD, CD36, FABP, ACC1 in macrophage (M1 and M2) are shown as relative fold to control normalized to β-actin. (L-O) mRNA expression of CPT1A, SCD, ACC1, FABP in Normal intestinal epithelium (NCM460) and colorectal cancer cells (HCT116, SW480, RKO and HT29) are shown as relative fold to control normalized to β-actin. Data represent means±SD. *p<0.05; **p<0.01; ***p<0.001.
