# Supplementary figures and images for: Single-cell transcriptome analysis reveals heterogeneity and convergence of the tumor microenvironment in colorectal cancer
Source: Front Immunol. 2023 Jan 4;13:1003419. doi: 10.3389/fimmu.2022.1003419 (PMC9845924; doi:10.3389/fimmu.2022.1003419)

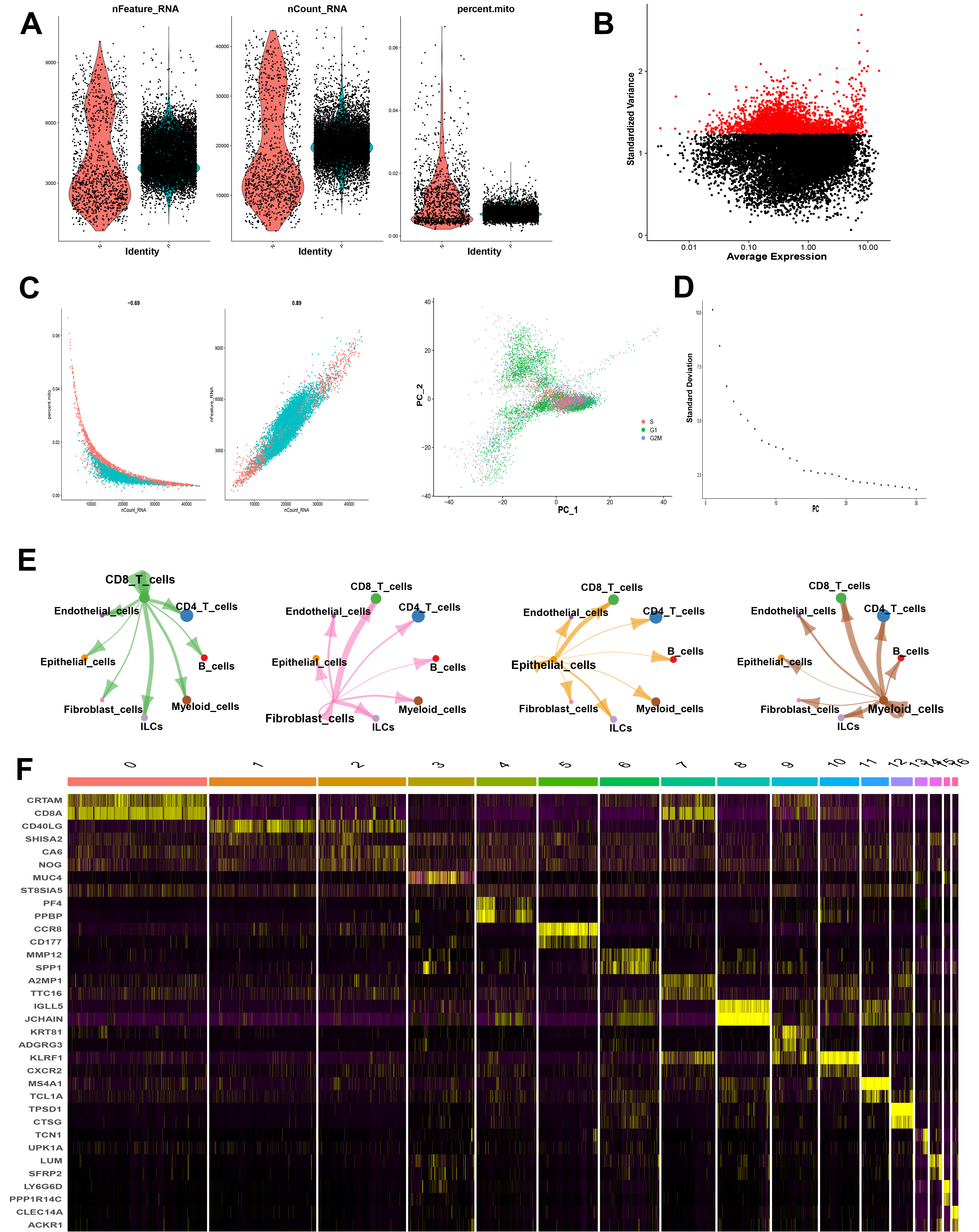

Supplement: Supplementary file 1 [file DataSheet_1.zip › Figure_S1.tif]

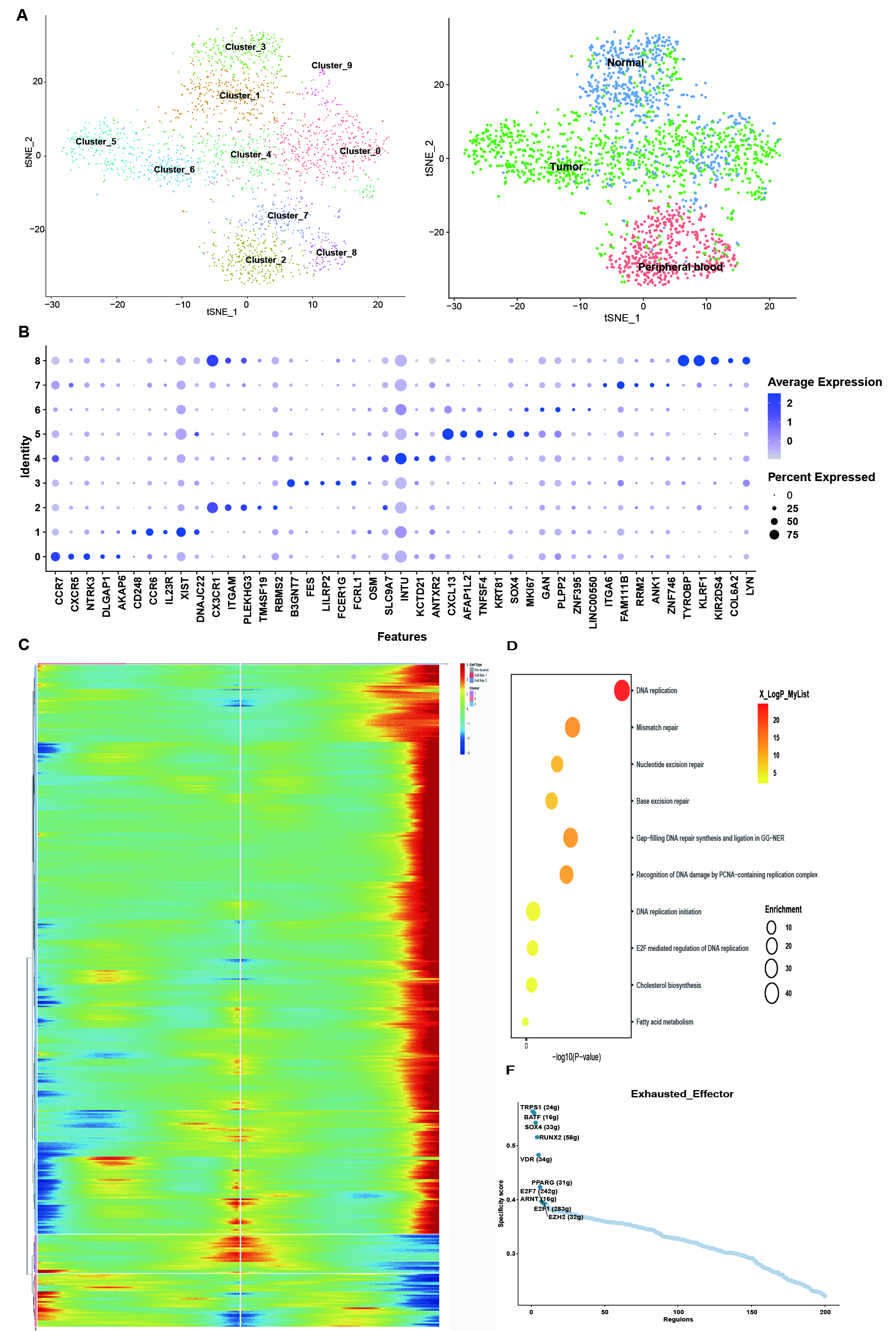

Supplement: Supplementary file 1 [file DataSheet_1.zip › Figure_S2.tif]

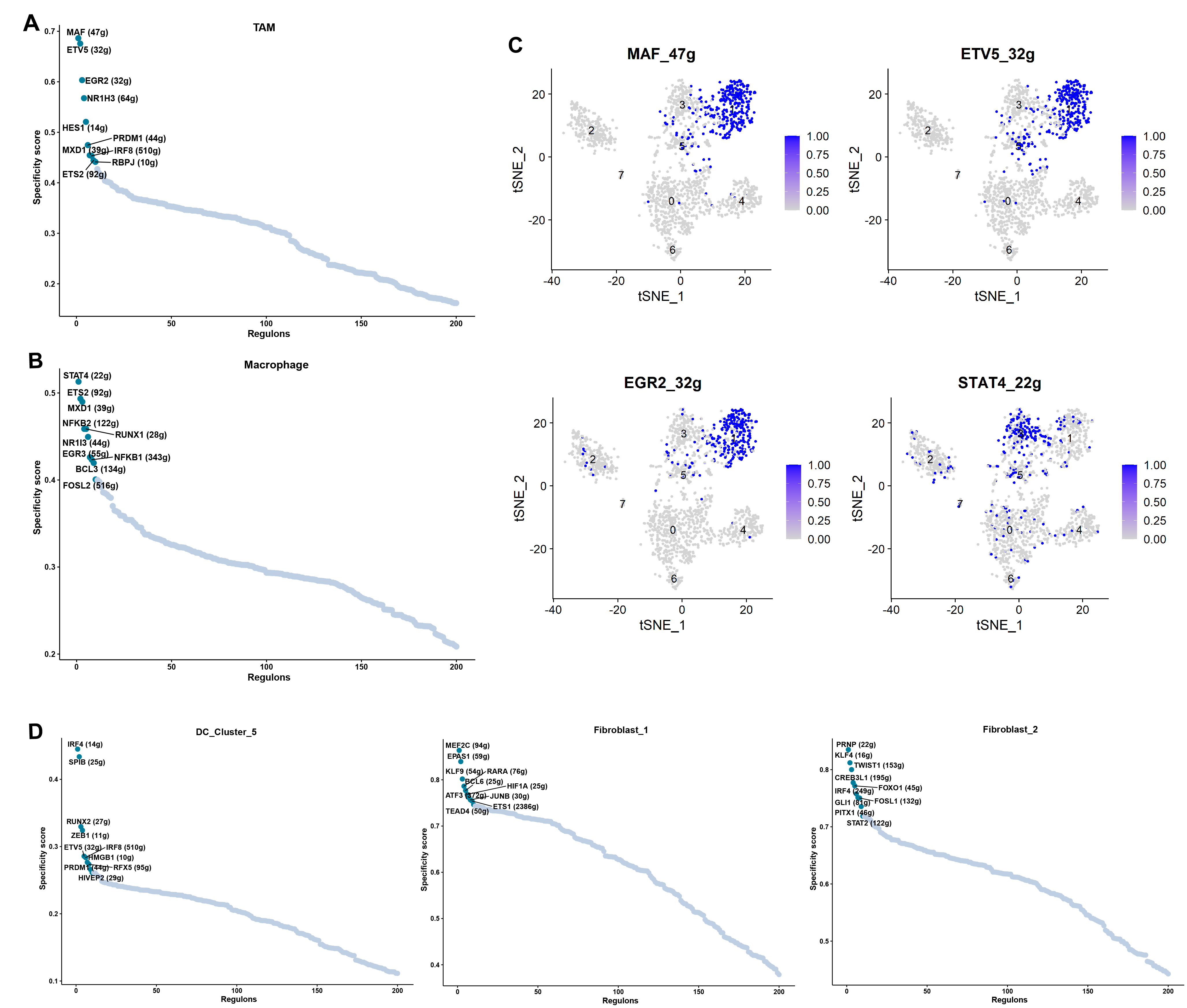

Supplement: Supplementary file 1 [file DataSheet_1.zip › Figure_S3.tif]

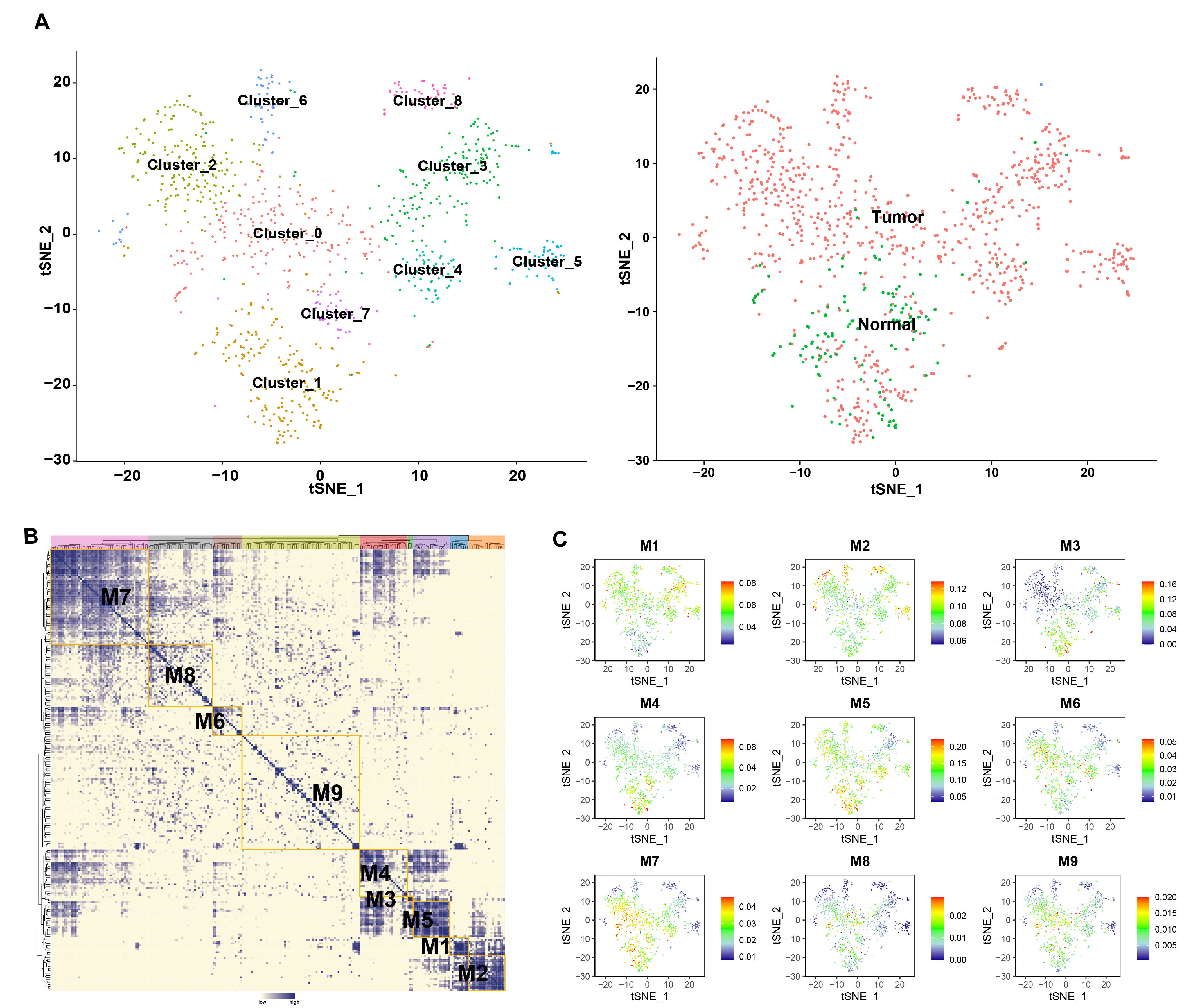

Supplement: Supplementary file 1 [file DataSheet_1.zip › Figure_S4.tif]

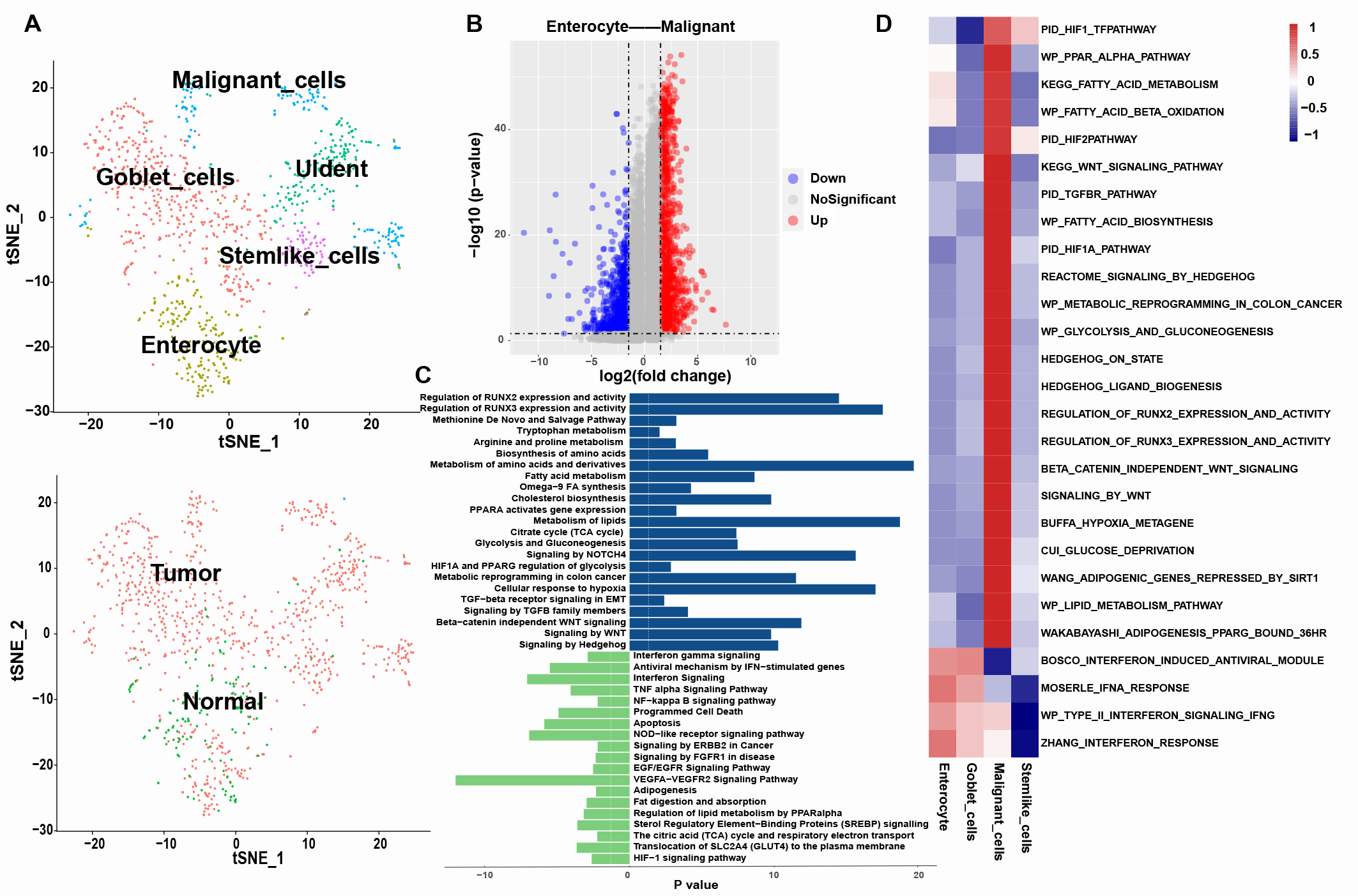

Supplement: Supplementary file 1 [file DataSheet_1.zip › Figure_S5.tif]

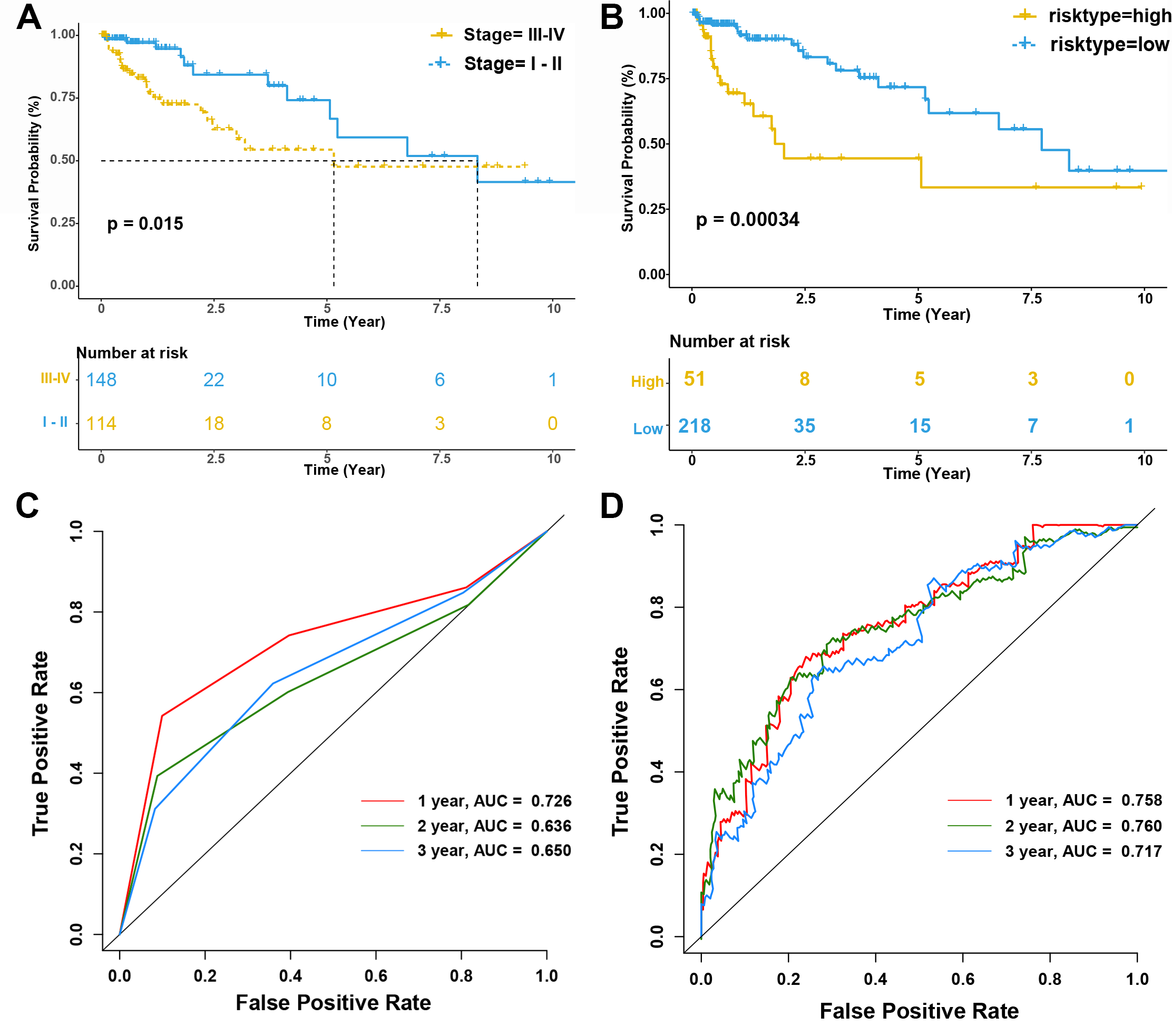

Supplement: Supplementary file 1 [file DataSheet_1.zip › Figure_S6.tif]

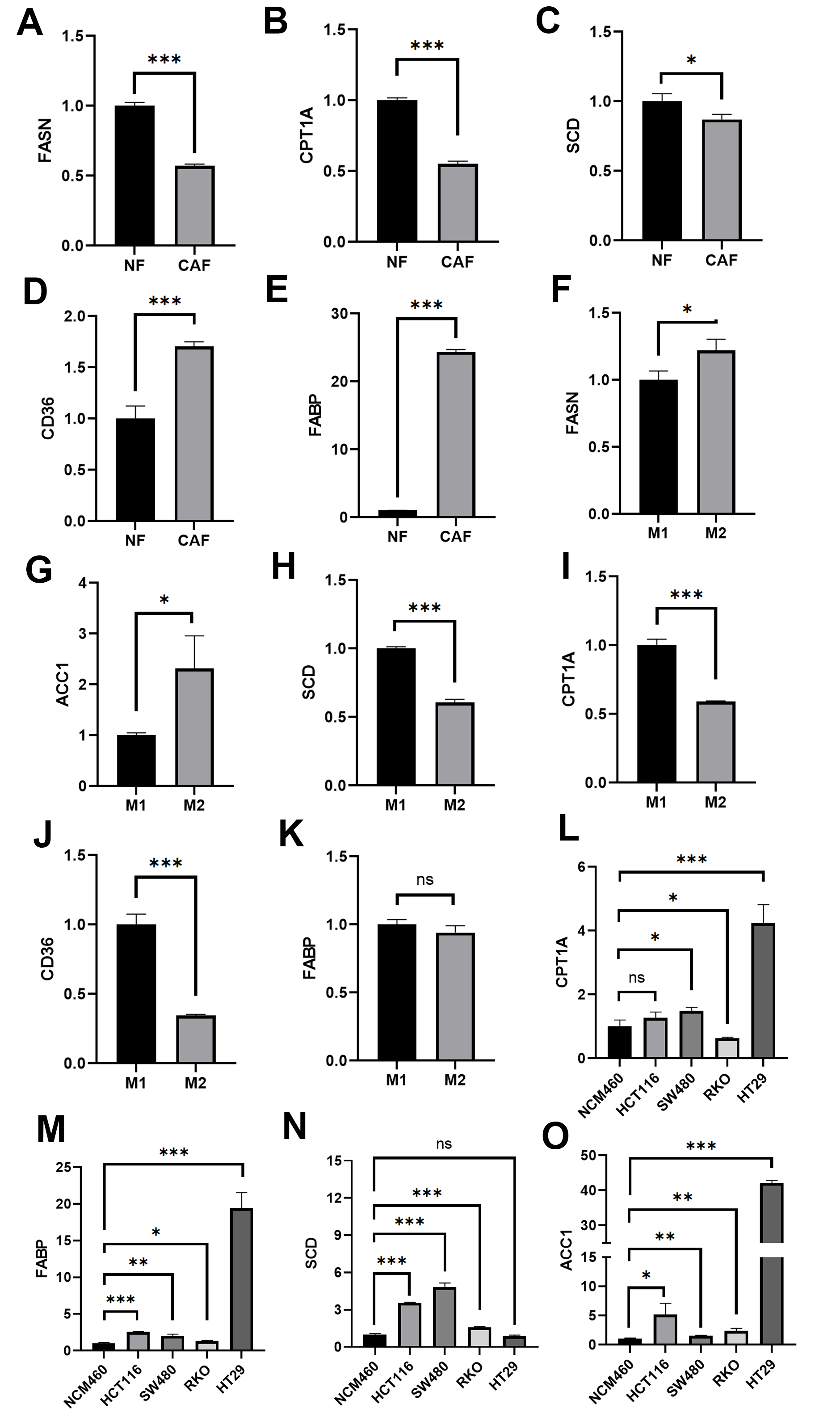

Supplement: Supplementary file 1 [file DataSheet_1.zip › Figure_S7.tif]
